# Comprehensive Identification and Expression Profiling of Circular RNAs During Nodule Development in *Phaseolus vulgaris*

**DOI:** 10.3389/fpls.2020.587185

**Published:** 2020-10-28

**Authors:** Zhihua Wu, Wen Huang, Erdai Qin, Shuo Liu, Huan Liu, Aleel K. Grennan, Hong Liu, Rui Qin

**Affiliations:** ^1^Hubei Provincial Key Laboratory for Protection and Application of Special Plant Germplasm in Wuling Area of China, College of Life Sciences, South-Central University for Nationalities, Wuhan, China; ^2^Key Laboratory of State Ethnic Affairs Commission for Biological Technology, College of Life Sciences, South-Central University for Nationalities, Wuhan, China; ^3^Biology Department, Worcester State University, Worcester, MA, United States

**Keywords:** circular RNA, *Phaseolus vulgaris*, regulation, nodulation, nitrogen fixation

## Abstract

Symbiotic nitrogen fixation by legume nodules provides an abundant nitrogen source for plants, and understanding this process is key for developing green agriculture. Circular RNA (circRNA), a type of endogenous RNA produced by reverse splicing of mRNA precursors, plays important regulatory roles in plants at the transcriptional and post-transcriptional levels. However, the relationship between circRNAs and legume–rhizobium is unknown. Here, we performed comprehensive identification and expression profiling of circRNAs during nodulation in common bean (*Phaseolus vulgaris*) compared to uninoculated roots of corresponding ages by constructing circRNA-seq and mRNA-seq libraries. We identified 8,842 high-confident circRNAs, 3,448 of which were specifically produced during symbiosis, with the highest number at the nitrogen-fixing stage. Significantly, more circRNAs were derived from exons than from intergenic regions or introns in all samples. The lengths and GC contents of the circRNAs were similar in roots and nodules. However, circRNAs showed specific spatiotemporal expression patterns during nodule and root development. GO and other functional annotation of parental genes of differentially expressed circRNAs indicated their potential involvement in different biological processes. The expression of major circRNAs during symbiosis is independent of parental genes’ expression to a certain degree, while expression of the remaining minor circRNAs showed positive correlation to parental genes. Functional annotation of the targeted mRNAs in the circRNA–miRNA–mRNA network showed that circRNAs may be involved in transmembrane transport and positive regulation of kinase activity during nodulation and nitrogen fixation as miRNA sponges. Our comprehensive analysis of the expression profile of circRNAs and their potential functions suggests that circRNAs may function as new post-transcriptional regulators in legume–rhizobium symbiosis.

## Introduction

The economically and ecologically important Leguminosae family, the third largest family of angiosperms, is widely distributed in diverse environments worldwide. Root nodule symbiosis is one of the most productive nitrogen-fixing systems. Approximately 88% of legume species form specialized symbiotic nitrogen-fixing nodules, which convert inorganic nitrogen in the atmosphere into organic ammonium via symbiosis with rhizobia. This process plays important roles in maintaining ecosystem function and sustainable agriculture (Doyle and Luckow, 2003). Nodulation, is a multistep process, triggered by rhizobial nodulation factors (NFs), and reactivates plant differentiated cortical cells (Crespi and Frugier, 2008). Common bean (*Phaseolus vulgaris* L., 2n = 22), an important nitrogen-fixing legume crop, is used as a model food legume due to its high protein, low fat, and high nutrient levels (Broughton et al., 2003). From the perspective of symbiosis, common bean is promiscuous in that it forms nitrogen-fixing nodules with a wide diversity of rhizobia from the *Rhizobium* and *Sinorhizobium* genera (Velazquez et al., 2001). Common bean is more closely related to soybean than to other model legumes such as the forage crops *Lotus japonicus* and alfalfa; common bean and soybean both belong to the phaseoloid group along with most other legume crops (Azani et al., 2017). Moreover, the genome size of the diploid common bean (∼587 Mb) is nearly half that of the paleopolyploid soybean (∼1,115 Mb), which underwent two rounds of whole-genome duplications (Schmutz et al., 2014). Therefore, common bean is an ideal model diploid for studying gene regulation and evolution of legume–rhizobium symbiosis in the phaseoloid group (McClean et al., 2008).

Multiple types of non-coding RNAs play important roles in root nodule symbiosis of the phaseoloid group via regulating expression of protein-coding genes. For example, increased AP2-miR172c improved nodule number and nitrogen fixation via targeting *AP2-1* mRNA in both common bean (Nova-Franco et al., 2015) and soybean (Wang et al., 2014). A total of 132 miRNAs and 1,984 phasiRNAs were identified in NF-induced root hairs and non-induced roots in common bean (Formey et al., 2016). In the interactions between rhizobia and soybean, a novel type of non-coding RNAs, tRFs (rhizobial tRNA-derived small RNA fragments), act as signaling molecules to regulate nodulation (Ren et al., 2019). Another type of endogenous non-coding RNA, circular RNA (circRNA), is produced by reverse splicing of mRNA precursors and has become an increasing focus of study in the field of non-coding RNA. At the post-transcriptional level, circRNAs act as ceRNAs (competing endogenous RNAs), functioning as “sponges” that associate with and sequester specific miRNAs to prevent them from interacting with their target mRNAs (Hansen et al., 2013). CircRNAs also participate in transcriptional regulation, interactions with RNA-binding proteins, and photosynthesis, thereby playing important roles in the plant life cycle (Jeck and Sharpless, 2014; Li et al., 2015; Lu et al., 2015; Lee et al., 2017). Functional analysis of circRNAs has demonstrated that they play important roles in regulating both biotic and abiotic stress responses. For example, circR5g05160 is involved in the immune response of rice against *Magnaporthe oryzae* and improves disease resistance (Fan et al., 2020). CircGORK (GUARD CELL OUTWARD RECTIFYING K^+^-CHANNEL) regulates the drought-stress response in *A. thaliana* (Zhang et al., 2019).

As a new organ, root nodules evolved from roots (Crespi and Frugier, 2008) in the nitrogen-fixation clade about 100 million years ago (Mya) (Werner et al., 2014). During the legume–rhizobium symbiosis and root development with corresponding ages, the characteristics of circRNAs and their specific expression profiling are unclear. Also, the regulatory relationship among the circRNAs, miRNA, and mRNA during symbiosis is also unknown. Here, we firstly identified and characterized the circRNAs and their expression pattern in the nodule development and corresponding roots combined with the real-time PCR validation. Then, we further investigated the potential “sponge” function of circRNAs through analysis of circRNA–miRNA–mRNA networks in nodule organogenesis and nitrogen fixation.

## Materials and Methods

### Plant Materials

Common bean (cultivar, Tianmadidou) seeds were sterilized with 95% ethanol for 1 min, followed by 0.1% HgCl_2_ for 15 min, and transferred to 0.8% water agar for germination at 23°C, 45% relative humidity in the dark for 48 h. One seedling was planted in each pot. The sand was cleaned many times to remove possible organic nutrients and sterilized after bottling. The plants were irrigated with Fahraeus nitrogen-free nutrient solution and grown under a 16 h/8 h photoperiod at 23°C and 45% relative humidity. When the first true leaf emerged, 2 ml of *Rhizobium tropici* CIAT899 (OD_600_ = 0.2) purchased from Culture Collection of China Agricultural University was used to infect the roots, and nutrient solution was added every 4–6 days. The roots (or nodules) at 2 cm below the stem from average 2 plants were collected on the 1st day (inoculated roots at stage I1), 10th day [mixtures of roots and nodules (undergoing nodule formation) at stage I10], and 21st day after inoculation (DAI) [only nodules (involved in nitrogen fixation) at stage I21]. As a control, uninoculated root samples from the same position as the inoculated tissues were collected during the corresponding periods of inoculation: day 1 (C1), day 10 (C10), and day 21 (C21), respectively (Supplementary Figure 1). Three biological replicates were taken from each sample at each time point for circRNA-seq and mRNA-seq. The samples were immediately frozen in liquid nitrogen and stored at −80°C.

### CircRNA and mRNA Library Construction and Sequencing

Total RNA was isolated from the samples using TRIzol reagent. The concentration and purity of each RNA sample were determined using a NanoDrop ND-1000. RNA integrity was assessed using an Agilent 2100 Bioanalyzer with a threshold of RIN > 7.0. For the circRNA library, approximately 5 μg of total RNA was subjected to ribosomal RNA depletion using a Ribo-Zero^®^ rRNA Removal Kit. The remaining RNA was treated with RNase R to remove linear RNAs and enrich for circRNAs (Yin et al., 2015). Following the removal of ribosomal RNA and linear RNA, the enriched circRNAs were fragmented using divalent cations at high temperature. The cleaved RNA fragments were reverse transcribed, and the resulting cDNA was used to synthesize U-labeled second-stranded DNA with *Escherichia coli* DNA polymerase I, RNase H, and dUTP. Adapters were ligated to the fragments, and size selection was performed using AMPureXP beads. Following treatment of the U-labeled second-stranded DNA with the heat-labile enzyme UDG, the ligated products were PCR amplified using the following cycling conditions: initial denaturation at 95°C for 3 min; 8 cycles of denaturation at 98°C for 15 s, annealing at 60°C for 15 s, and extension at 72°C for 30 s; final extension at 72°C for 5 min. The average insert size for the final cDNA library was 300 bp (± 50 bp). For mRNA library, mRNA was purified and enriched with magnetic beads of oligo (dT) to remove ribosomal RNAs. After preparation of cDNA, the remaining procedures were similar to those for the circRNA library. Finally, we performed paired-end sequencing for circRNA and mRNA libraries on the Illumina NovaSeq 6000 platform following the vendor’s recommended protocol.

### Identification and Characterization of CircRNAs

Cutadapt (Martin, 2011) was used to remove reads containing adaptor contamination, low-quality bases, and undetermined bases. Sequence quality was then verified using FastQC (Andrews, 2010). BWA (Li and Durbin, 2009) and STAR (Dobin et al., 2013) were used to map the reads to the common bean genome (version, Pvulgaris_442_v2.1) in Phytozome. CIRI2 (Gao et al., 2015) and CIRCexplorer (Dong et al., 2019) were used to identify back-splicing reads, followed by *de novo* assembly of the mapped reads to circRNAs. Only circRNAs identified by both programs were considered to be valid candidate circRNAs, and the parental genes that produced corresponding circRNAs were identified by CIRI2.

### Validation of CircRNAs and Quantitative Reverse-Transcription PCR (qRT-PCR)

Total DNA was extracted from these samples using the CTAB method (Stewart and Via, 1993) for a negative control test. Total RNA was extracted using TRIzol reagent, reversed transcribed to cDNA, and digested with DNase I. Divergent and convergent primers for both DNA and cDNA of each sample were designed to verify the authenticity of the identified circRNAs. PCR amplification, gel electrophoresis detection, and sequencing of the PCR products with three replicates for each primer were then conducted. Divergent and convergent primers were designed to test the validity of circRNAs, and the reverse splices connecting the circRNAs were further confirmed by Sanger sequencing. The qRT-PCR primers were designed to quantify the expression of circRNAs. The expression level of circRNAs was standardized based on the expression of the endogenous linear *actin* (*Phvul.008G011000*) gene from common bean, with three independent replicates performed for each experiment.

### Differential Expression of CircRNAs and Functional Enrichment Analysis of Their Parental Genes

To evaluate mRNA expression, HISAT2 (Pertea et al., 2016) was used to align clean sequencing reads to the reference genome of common bean, and StringTie v1.3.3b (Pertea et al., 2015) was used to calculate the FPKM (Fragments Per Kilobase per Million) value for each gene in these samples. Differentially expressed mRNAs (DEmRNAs) were identified using DESeq2 (Love et al., 2014), with the following threshold, | log_2_ (fold change)| > 1 and adjusted *p* < 0.05. To evaluate the expression levels of circRNAs, we normalized the back-spliced reads by read length and number of mapped reads (Spliced Reads Per Billion Mappings, denoted as SRPBM), which permits quantitative comparison of back splicing from different RNA-seq data. Differentially expressed circRNAs (DEcircRNAs) were identified using edgeR (version 3.24.0) (Robinson et al., 2010) with the significance threshold set as | log_2_ (fold change)| > 1 and *p* < 0.05. AgriGO v2.0 (Tian et al., 2017) and KOBAS 2.0 (Xie et al., 2011) were used to analyze the enriched GO categories and KEGG pathways of parental genes of DEcircRNAs, respectively. The threshold for the enrichment analysis was set to *p* < 0.05.

### Prediction of CircRNA–miRNA–mRNA Networks

To investigate whether the DEcircRNAs function as ceRNAs in combination with DEmRNAs during symbiosis, we collected and removed redundant miRNA sequences of common bean from miRBase (Kozomara and Griffiths-Jones, 2014) and two sets of microRNA data from published literatures (Peláez et al., 2012; Formey et al., 2016). Based on the circRNAs and mRNAs identified in our study, we constructed circRNA–miRNA–mRNA networks at both stages of the nodule formation and nitrogen fixation. The methods were as follows: (1) determine the potential binding relationship between a circRNA and miRNA, a miRNA and mRNA using psRobot_tar (Wu et al., 2012), psRNATarget (Dai and Zhao, 2011), and TAPIR (Bonnet et al., 2010), simultaneously; (2) keep the binding relationships of circRNA–miRNA and miRNA–mRNA predicted from at least two software above; (3) select the coregulated miRNA-binding DEcircRNAs and DEmRNAs for possible regulatory circRNA–miRNA–mRNA relationship; (4) visualize the circRNA–miRNA–mRNA regulatory networks using Cytoscape v3.7.2 software (Shannon et al., 2003) to display the potential associations between circRNAs, miRNAs, and mRNAs.

## Results

### Genomic and Transcriptional Features of CircRNAs in Nodule and Root Development

**FIGURE 1:**
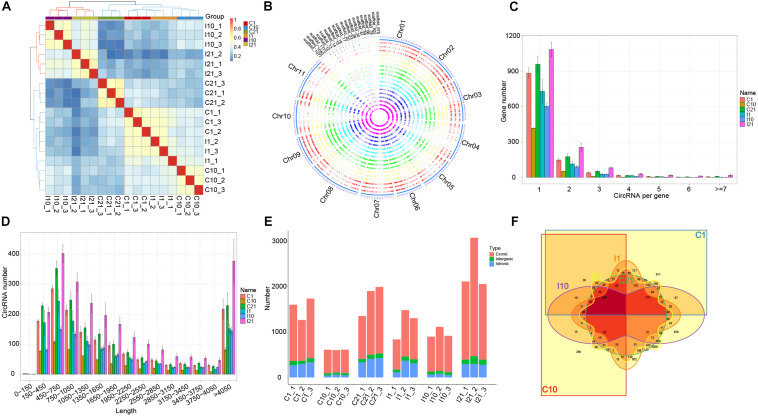
Characteristics of circRNA in roots and nodules of common bean. **(A)** Correlation coefficients for biological repeats of the six treatment groups. **(B)** Circos diagram showing the distribution and expression levels of circRNAs identified in the common bean genome. **(C)** Number of circRNAs produced per gene. **(D)** Length distribution of circRNAs. **(E)** Number of exonic circRNAs, intergenic circRNAs, and intronic circRNAs in each sequenced sample. **(F)** Venn diagram showing the number and distribution of circRNAs in the six treatment groups.

After trimming adaptor sequences and filtering low-quality reads, we obtained 1,459,179,756 high-quality reads from a total of 18 samples (I1, I10, I21, C1, C10, and C21, each with three independent replicates) via circRNA-seq, with an average error rate of 0.0124% (Supplementary Table 1). Based on the expression of circRNAs, correlation and principal component analysis of the 18 samples indicated that the independent triplicates under each treated condition were similar. Meanwhile, the 18 samples were divided into two clusters, indicating that the expression pattern of circRNAs in nodules (I10 and I21) are different from that in roots (Figure 1A and Supplementary Figure 2A). A total of 8842 high-confident circRNAs produced from 4155 parental genes were identified, most with GC contents of 25–35% (Supplementary Figure 2B and Supplementary Table 2). There were different numbers of circRNAs on each chromosome and scaffold, with the most circRNAs on chromosome 3 (Supplementary Figure 2C). Overall, there was low expression of circRNAs from all samples in all chromosomes and scaffolds (Figure 1B). Most of the parental genes produced only one circRNA, although a few produced multiple circRNAs (Figure 1C). CircRNAs with lengths from 150 to 1,050 bp were the most common, and circRNAs of almost all lengths were more abundant in nitrogen-fixing nodules (I21) than in the other samples (Figure 1D), indicating that more circRNAs were expressed in the nitrogen-fixing process.

An analysis of the genomic sources of circRNAs showed that the number of circRNAs in exons (69.80–87.23%) was significantly higher than the number of circRNAs in intergenic regions and introns, indicating that circRNAs of both roots and nodules of common bean are primarily derived from exons. A comparison of the number of circRNAs from the six groups (I1, I10, I21, C1, C10, and C21) showed that circRNA formation in roots and nodules exhibited spatiotemporal specificity. The highest number of circRNAs was present in I21 and the lowest was in C10 (Figure 1E). We detected 5,394 circRNAs in uninoculated roots (C1, C10, and C21) and 6342 in post-inoculated roots and nodules (I1 I10, and I21), of which 2,451, 2,027, and 4,634 circRNAs were identified in I1, I10, and I21, respectively. Of these circRNAs, 2,894 were common to both inoculated and uninoculated tissues, 3,448 were uniquely present after inoculation, and 2,500 were unique in uninoculated roots, indicating that the interaction with rhizobia led to more kinds of circRNAs compared to root development without inoculation (Figure 1F and Supplementary Table 2).

**FIGURE 2:**
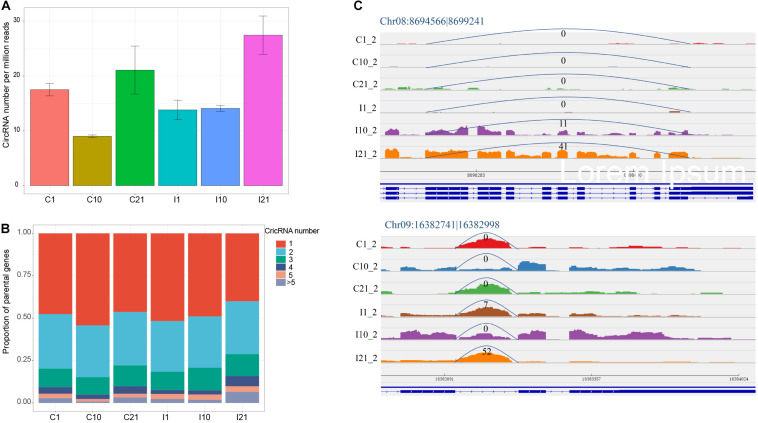
CircRNAs with expressed diversity during root and nodule development. **(A)** Normalized circRNA numbers per million high-quality sequencing tags. **(B)** Proportion of parental genes deriving different numbers of circRNAs for each developmental stage. **(C)** Higher expression of the exonic circRNA (Chr08:8694566| 8699241) and intronic circRNA (Chr09:16382741| 16382998) in I21 than in other five samples. The numbers at the arc lines indicate the numbers of junction reads.

The normalized number of circRNAs per million high-quality sequencing reads confirmed that I21 had the most circRNAs and that C10 had the fewest (Figure 2A). To determine the potential reasons for the increase in circRNA diversity in I21, we analyzed the proportions of genes producing different number of circRNAs. There were higher proportions of genes with 4 and > 5 circRNAs in I21 than in the other groups (Figure 2B), indicating that the total number of circRNAs is correlated to the proportion of parental genes producing more circRNA numbers per gene. Meanwhile, for one circRNA from one gene locus, the specific expression in mature nodules contributed to the increased numbers of circRNAs. For example, the expression of Chr08:8694566 |8699241 increased throughout nodule development, and Chr09:16382741| 16382998 was expressed at significantly higher levels in nodules during maturation (I21) than in other samples (Figure 2C). Here, we obtained a comprehensive landscape of genomic origins, length distribution, GC contents, and expression profiling of circRNAs during nodule and root development.

**FIGURE 3:**
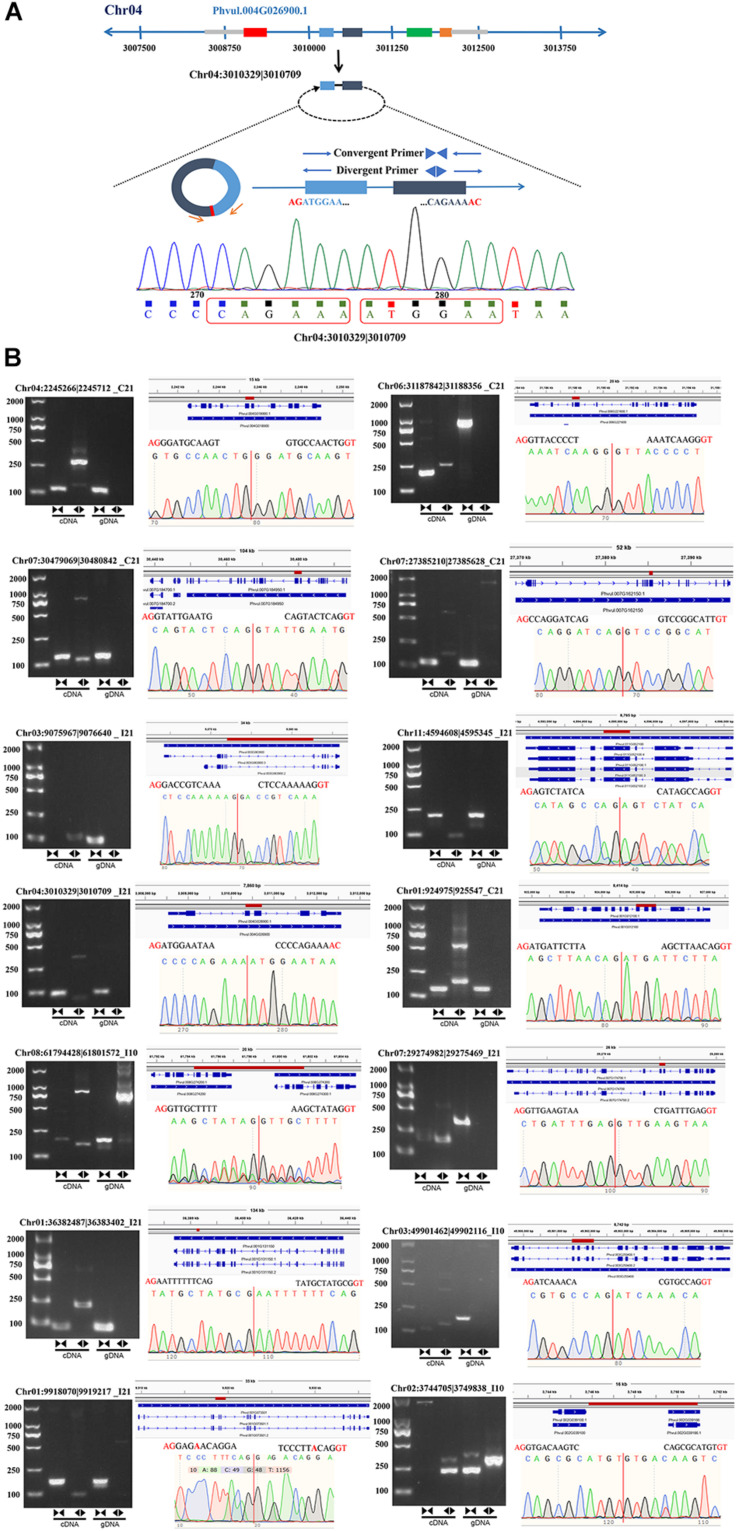
Experimental verification of the stable expression of circRNAs in common bean. **(A)** An example (Chr04:3010329| 3010709) illustrating the validation strategy. **(B)** Representative circRNAs were verified by PCR amplification using divergent and convergent primers.

To further verify the reliability of the circRNA-seq data, we designed divergent and convergent primers to amplify the reverse splices of genomic DNA (gDNA) and cDNA. Unlike the convergent primers, the divergent primers could only be used to amplify products from cDNA of circRNAs and not from gDNA (Figure 3A). We randomly selected 16 circRNAs, 14 of which were successfully amplified (Figure 3B and Supplementary Table 3), including 11 exonic circRNAs, 2 intergenic circRNAs, and 1 intronic circRNA. These results indicate that most of our newly identified circRNAs in roots and nodules are genuine.

### CircRNAs Are Differentially Regulated During Early Inoculation, Nodule Organogenesis, and Nitrogen Fixation

**FIGURE 4:**
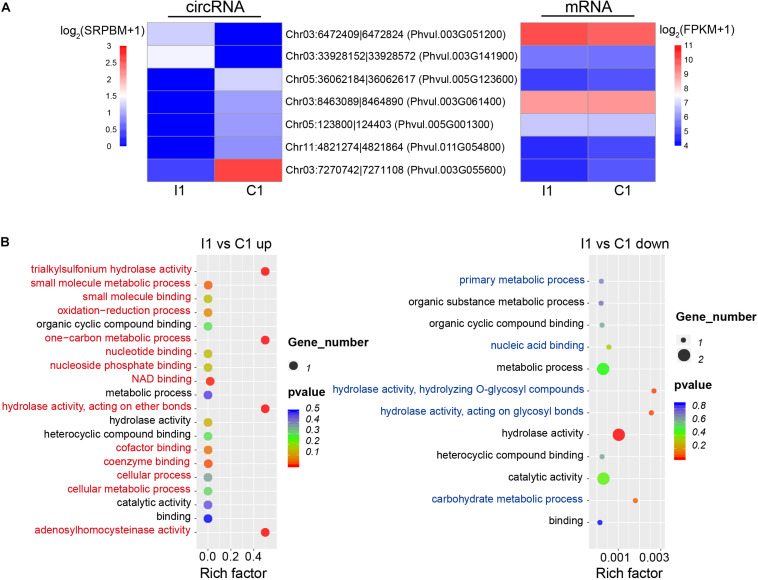
Expression patterns and potential functions of DEcircRNAs during early inoculation. **(A)** Expression patterns of DEcircRNAs (left) and parental genes (right). **(B)** GO annotations of upregulated and downregulated DEcircRNAs; red and blue indicate unique GO categories of upregulated and downregulated genes, respectively, and black indicates common GO categories. Rich factor is defined as the ratio between the number of DEcircRNAs’ parental genes annotated to KEGG terms and the number of the background DEcircRNAs’ parental genes annotated to KEGG terms.

Changes in the expression levels of the circRNAs and their specific expression patterns suggest that they may play important roles in different biological processes during nodulation. Compared with uninoculated root hairs, inoculated root hairs showed deformation (Supplementary Figure 1), which is also reported in the previous study (Formey et al., 2016). Seven DEcircRNAs were identified during the early stage of inoculation (I1 vs. C1), with two upregulated intronic (Chr03:6472409| 6472824, Chr03:33928152| 33928572) and five downregulated circRNAs (three were intronic, one was exonic, and one was intergenic). There was no significant coregulation observed based on the abundance of circRNAs and parental gene expression during early inoculation stage (Figure 4A). GO annotation of these parental genes showed that the upregulated and downregulated circRNAs participated in different biological functions, respectively, such as unique process of oxidation–reduction for parental genes (*Phvul.003G051200* coding the S-adenosyl-L-homocysteine hydrolase) of upregulated circRNA (Chr03:6472409| 6472824) (Figure 4B).

**FIGURE 5:**
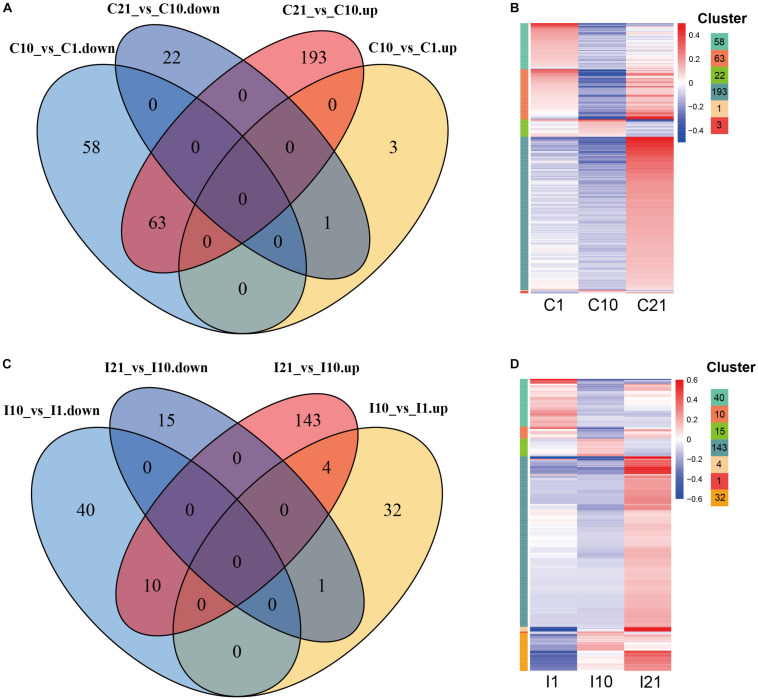
Expression patterns of DEcircRNAs during root and nodule development. **(A)** Up- and downregulated root-specific DEcircRNAs during consecutive periods during root development. **(B)** Expression patterns of root-specific DEcircRNAs during root development. The left color bars of the heatmap represent the DEcircRNAs from each part with numbers in **(A)**. **(C)** Up- and downregulated nodule-specific DEcircRNAs during consecutive periods during nodule development. **(D)** Expression patterns of nodule-specific DEcircRNAs during nodule development. The left color bars of the heatmap represent the DEcircRNAs from each part with numbers in **(C)**. The length of each color bar represents the number of the DEcircRNAs in each part of **(A,C)**, also shown on the top right.

To explore the potential functions of DEcircRNAs in nodule formation and nitrogen fixation, we compared DEcircRNAs during root and nodule development, and identified DEcircRNAs that were unique to roots or nodules. After filtering for co-upregulated and co-downregulated DEcircRNAs in both inoculated and uninoculated tissues, unique DEcircRNAs (245) were identified from nodules compared to DEcircRNAs in uninoculated roots (340) during the corresponding period (Figure 5). In comparisons of circRNAs on day 21 vs. day 10 (I21 vs. I10, C21 vs. C10), both nodules (I21, 143) and roots (C21, 193) contained more upregulated DEcircRNAs on day 21. In comparisons of circRNAs on day 10 vs. day 1 (I10 vs. I1, C10 vs. C1), the number of upregulated DEcircRNAs was much higher in nodules (32) than in roots (3), suggesting that more circRNAs are possibly involved in regulating nodule vs. root organogenesis. Compared to nodule organogenesis (32 DEcircRNAs), more upregulated DEcircRNAs (143) were regulated at the nitrogen-fixing stage. Compared with uninoculated roots, there were fewer unique downregulated but more unique upregulated DEcircRNAs in young nodules at the stage of nodule formation, while there were fewer unique DEcircRNAs in nitrogen-fixing nodules (Supplementary Figure 3).

**FIGURE 6:**
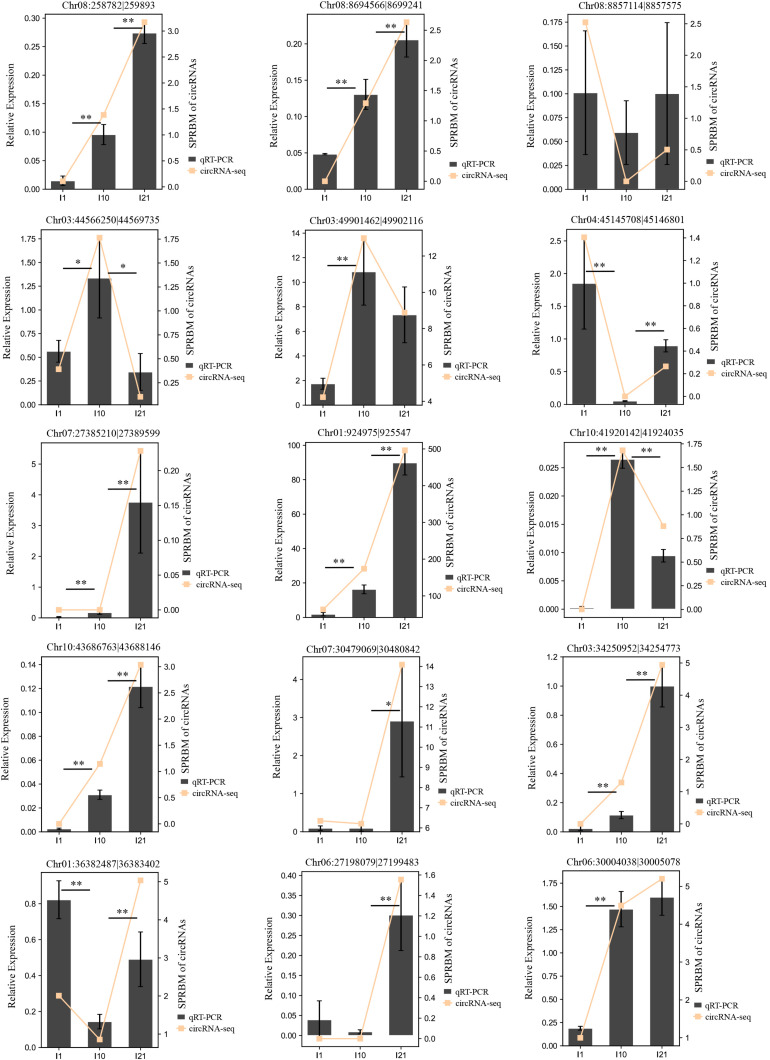
Confirmation of the expression patterns of the DEcircRNAs by qRT-PCR. *Actin* gene (*Phvul.008G011000*) was used as a reference gene. The error bars indicate the standard deviation of three replicates. Asterisks indicate a significant difference, as determined by Student’s *t-*test (^∗^*P* < 0.05; ^∗∗^*P* < 0.01).

To verify the reliability of the circRNA-seq expression profiles, we randomly selected 15 DEcircRNAs and subjected them to expression analysis by qRT-PCR (Figure 6 and Supplementary Table 4). The expression changes during nodulation and nitrogen fixation revealed by qRT-PCR were basically consistent with the results of circRNA-seq, indicating that the detection method used in this experiment is reliable and highly accurate.

### Parental Genes of DEcircRNAs Are Associated With Different Biological Processes for Nodule Organogenesis and Nitrogen Fixation

**FIGURE 7:**
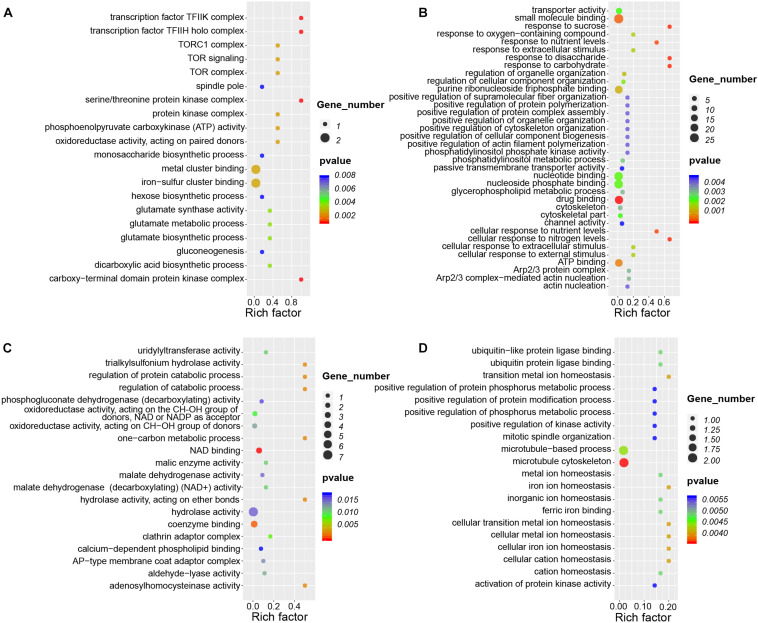
GO enrichment analysis of circRNAs during root and nodule development. **(A)** Enriched GO categories of 32 DEcircRNAs that are specifically upregulated during nodule organogenesis. **(B)** Enriched GO categories of 143 DEcircRNAs that are specifically upregulated during nitrogen fixation. **(C)** Enriched GO categories of 40 DEcircRNAs that are specifically downregulated during nodule organogenesis. **(D)** Enriched GO categories of 15 DEcircRNAs that are specifically downregulated during nitrogen fixation. Rich factor is defined as the ratio between the number of DEcircRNAs’ parental genes annotated to KEGG terms and the number of the background DEcircRNAs’ parental genes annotated to KEGG terms.

CircRNAs *cis*-regulate the expression of their parental genes, thus playing biological roles related to those of their parental genes (Li et al., 2015, 2018). To investigate the potential functions of circRNAs during nodule development, we conducted GO and KEGG analysis of parental genes of these circRNAs present during nodule formation and nitrogen fixation. During nodule formation, the parental genes of DEcircRNAs were involved in many biological processes (Figure 7A). For example, the parental genes of seven upregulated circRNAs were enriched in the GO categories “iron-sulfur cluster binding” (GO:0051536), “glutamate synthase activity” (GO:0006537) involving in modification of nitrogen metabolism in nodules (Harrison et al., 2003), and “gluconeogenesis” (GO:0006094). Iron–sulfur clusters are essential cofactors of nitrogenase in nitrogen fixation (Britt et al., 2020). Nodule formation and nitrogen fixation are energy-consuming processes (Mortier et al., 2012), gluconeogenesis may lay a foundation of energy supply. Therefore, these results indicated that the circRNAs may be important for nodule development.

During nitrogen fixation, parental genes of upregulated DEcircRNAs were significantly enriched in these GO terms of “response to sucrose” (GO:0009744), “response to disaccharide” (GO:0034285), and “cellular response to nitrogen levels” (GO:0043562) (Figure 7B), which were different from nodule formation. Meanwhile, there were little overlapped enriched GO terms between nodule organogenesis and nitrogen fixation. The enrichment of downregulated DEcircRNAs in the GO terms of nodule organogenesis and nitrogen fixation also points to their potentially unique functions (Figures 7C,D). KEGG pathway analysis revealed that the parental genes were enriched in different pathways for nodule formation and nitrogen fixation. For example, “cell cycle” (ko04110), “glycosaminoglycan degradation” (ko00531), and “glycolysis/gluconeogenesis” (ko00010) were enriched during nodule formation, and “tight junction” (ko04530) and “ABC transporters” (ko02010) were enriched during nitrogen fixation (Supplementary Figure 4). These differentially enriched GO terms and KEGG pathways point to potentially differential roles of circRNAs during nodule formation and nitrogen fixation.

### Expression of the Minor CircRNAs Is Positively Correlated to Parental Gene Expression During Nodule Organogenesis and Nitrogen Fixation

**FIGURE 8:**
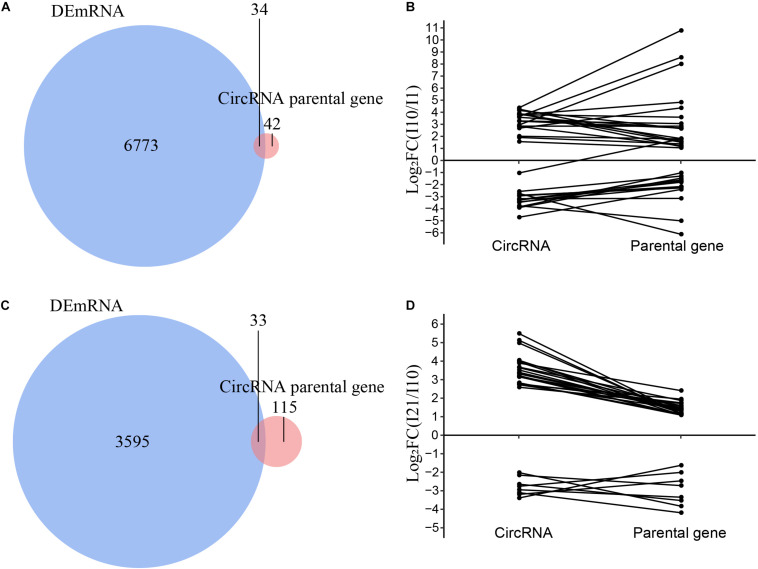
Correlation analysis of the expression of circRNAs and their parental genes. **(A)** Venn diagram of the parental genes of DEcircRNAs and DEmRNAs during nodule organogenesis. **(B)** Correlation analysis of the parental genes of DEcircRNAs and DEmRNAs during nodule organogenesis. **(C)** Venn diagram of the parental genes of DEcircRNAs and DEmRNAs during nitrogen fixation. **(D)** Correlation analysis of the parental genes of DEcircRNAs and DEmRNAs during nitrogen fixation.

To analyze the relationships between DEcircRNAs and expression of their parental genes, we identified the intersections of the parental genes of DEcircRNAs and all differentially expressed mRNAs (DEmRNAs). The numbers of DEcircRNAs and DEmRNAs during nodule formation and nitrogen fixation were significantly different (Supplementary Figure 5). Meanwhile, only 34 and 33 parental genes were also identified as DEmRNAs in nodule formation and nitrogen fixation, respectively, suggesting that the majority of DEcircRNAs were regulated independently of their parental genes (Figures 8A,C). The correlation between DEcircRNAs and DEmRNAs of their parental genes suggested that the remaining minor DEcircRNAs were mainly positively correlated during both nodule organogenesis and nitrogen fixation in common bean (Figures 8B,D). These minor DEcircRNAs with positive correlations to parental genes may be upregulated as a consequence of enhanced transcription of the parental genes, which is also reported in other plants before (Ye et al., 2015).

### Some DEcircRNAs May Function as MiRNA Sponges During Nodule Organogenesis and Nitrogen Fixation

**FIGURE 9:**
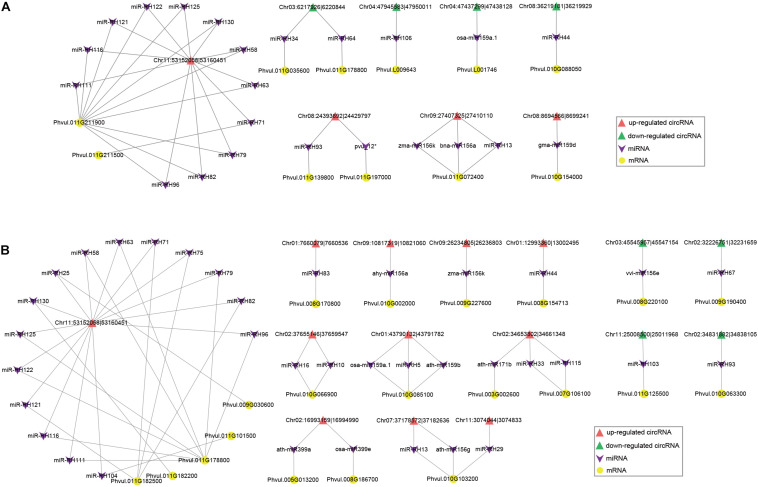
The circRNA–miRNA–mRNA regulatory networks during nodule organogenesis **(A)** and nitrogen fixation **(B)**. Upregulated and downregulated DEcircRNAs and DEmRNAs are shown with different shapes in the figures.

To investigate whether circRNAs target miRNAs to post-transcriptionally regulate gene expression during nodule development, we examined the miRNA binding capacity of circRNAs based on publicly available miRNA data and identified 1,272 (14.4%) circRNAs with 229 possible miRNA binding sites (Supplementary Table 5). As miRNA sponges, upregulated circRNAs can adsorb miRNAs and thus reduce miRNAs’ expression, and further enhance the regulation of miRNAs’ targeted genes. Our predicted circRNA-binding miRNAs, such as miR2111 (Tsikou et al., 2018), miR156 (Wang et al., 2015), miR166 (Boualem et al., 2008), and miR172 (Wang et al., 2014), were reported to be involved in regulation of nodule numbers in *L. japonicus*, *Medicago truncatula*, and soybean. During nodule formation, 23 miRNAs have predicted binding relationships with 8 DEcircRNAs (5 downregulated and 3 upregulated DEcircRNAs) and 11 DEmRNAs, respectively (Figure 9A and Supplementary Table 6). GO and KEGG analysis showed that these DEmRNAs were enriched in multiple biological and metabolic processes (Supplementary Tables 7, 8). For example, upregulated DEmRNAs were enriched in “transmembrane transport” (GO:0055085) and “ABC transporters” (ko02010). An upregulated DEcircRNA, Chr11:53152058 | 53160451, possibly functions as a sponge of 11 miRNAs, which targets its own parental gene (*Phvul.011G211900*). The optimal homolog of *Phvul.011G211900* was annotated as a Fe-citrate transporter important for symbiotic nitrogen fixation in *M. truncatula* (*Medtr6g004220*). We identified 33 miRNAs potentially having targeted relationships with 15 DEcircRNAs (4 downregulated and 11 upregulated DEcircRNAs) and 20 DEmRNAs during nitrogen fixation (Figure 9B and Supplementary Table 9). Compared with the stage of nodule organogenesis, there are more upregulated DEcircRNAs regulated at the nitrogen-fixing stage. GO and KEGG analysis showed that during nitrogen fixation, the circRNA–miRNA–mRNA network may be involved in multiple important processes, such as “positive regulation of kinase activity” (GO:0033674), “sulfate transport” (GO:0008272), and “glycolysis/gluconeogenesis” (ko00010) (Tables 10, 11). Sulfate transporter was essential for symbiotic nitrogen fixation (Krusell et al., 2005). In the circRNA–miRNA–mRNA network during symbiotic nitrogen fixation, a sulfate transporter (Phvul.008G170800) was possibly targeted by miR-RH83, which was regulated by Chr01:7660079| 7660536.

## Discussion

As a relatively new organ, nodules originated around 100 Mya (Werner et al., 2014), whereas roots arose about 416–360 Mya when plants colonized land (Kenrick and Strullu-Derrien, 2014). Therefore, there are common and specific regulatory genes involved in nodules and roots (Soyano et al., 2019). In the comprehensive investigation of circRNAs in developing roots and corresponding nodules using deep sequencing reads, we observed no significant differences in circRNA types, lengths, GC contents, and chromosome distribution between uninoculated roots and inoculated roots/nodules, indicating that the circRNA formation mechanism may be conserved for the two organs. Compared to young nitrogen-prefixing nodules and mature nitrogen-fixing nodules, the fewest DEcircRNAs were identified during the early inoculated stage, suggesting that more complex regulation of circRNAs occurs during nodule formation and nitrogen fixation. Nodules produced more unique DEcircRNAs than roots, especially at the stage of nitrogen fixation. More DEcircRNAs were present in young nodules (I10 vs. I1) than in the corresponding roots (C10 vs. C1), indicating that the new organ organogenesis may have greater DEcircRNA involvement.

CircRNAs are often involved in regulating the expression of their parental genes in humans, plants, and animals. For example, some circRNAs directly act on phosphorylated RNA polymerase II or bind to the promoter regions of parental genes to regulate their expression (Zhang et al., 2013; Li et al., 2015). In rice, however, the expression of most circRNAs is positively correlated with that of their parental genes (Ye et al., 2015), and some circRNAs also act as negative regulators of their parental genes (Lu et al., 2015). In tea plants (*Camellia sinensis*), the expression levels of some circRNAs and their parental mRNAs are positively correlated, whereas others are negatively correlated (Tong et al., 2018). In the current study, most of the parental genes of DEcircRNAs were not themselves differentially expressed, indicating that these circRNAs might function independently of their parental genes. When both the parental genes and their circRNAs were differentially expressed, almost all of the DEcircRNAs were positively correlated with the expression of their parental genes with one exception, Chr01:34214223| 34218537, which was negatively correlated with the expression of its parental gene (*Phvul.001G123900*). However, this gene produced three different circRNAs, including two whose expression (Chr01:34208805| 34212597 and Chr01:34214223| 34216626) was positively correlated with its expression. The certain circRNAs from one gene locus have the opposite regulation, suggesting their potentially regulatory complexity.

Enrichment analysis of DEcircRNAs’ parental genes pointed to their possibly unique roles in early inoculation, nodule formation, and nitrogen fixation. During the rhizobial infection period, increased production of reactive oxygen species (ROS) is a common plant defense response (Gourion et al., 2015). During the early stage of rhizobia inoculation, high levels of ROS are produced, functioning as signaling molecules in the establishment of the nitrogen-fixing legume–rhizobium symbiosis (Ebertová, 1959). ROS is required for optimal nodule functioning observed in common bean (Arthikala et al., 2014). Sulfenylation of protein cysteine residues modified by ROS is an important post-translational modification, and modulates various biological processes during legume–rhizobium symbiosis. In our study, one upregulated circRNA (Chr03:6472409| 6472824 from its parental gene, *Phvul.003G051200*) was involved in oxidation–reduction at 1 DAI. The optimal homolog of *Phvul.003G051200* is *Medtr8g083090* (annotated as S-adenosyl-L-homocysteine hydrolase), which is sulfenylated by ROS 2 days after inoculation during *M. truncatula*–*Sinorhizobium meliloti* symbiosis (Oger et al., 2012).

During the last 20 years, nearly 200 functional genes have been reported in legume nodulation and symbiotic nitrogen fixation (Roy et al., 2020). In our study, 55 genes of these reported genes can produce circRNAs in roots and nodules of common bean, 36 symbiotic genes (65.5%) of which only occurred in inoculated roots and nodules. During nodulation and nitrogen fixation, circRNAs from *LjSIP1* only occurred in inoculated roots (I1); *LjPHYB*, *LjSYMRK*, *MtNF-YA1*, *MtSYP132*, and *MtVPE* only occurred in nodule formation (I10); *GmbHLHm1*, *GmG*β*1/GmG*β*2/GmG*β*3/GmG*β*4*, *LjARPC1*, *LjBZF*, *LjCLC1*, *LjGLE1*, *LjHIP*, *LjNIN*, *LjPIR*, *MsNADH-GOGAT*, *Mt*γ*ECS*, *MtARP3*, *MtDME*, *LjLIN*, *MtPEN3*-like, *MtPHO2*-like, *MtRDN1*, *PvPI3K*, and *MtSymCRK* only occurred in nitrogen-fixing nodules (I21). The remaining symbiotic genes producing circRNAs occurred in one more stage (Supplementary Table 2). For example, MtARP3 (Phvul.002G253600), a component of the actin nucleation complex, plays indispensable roles in the development of symbiosomes, the vesicles that house the nitrogen-fixing bacteria within nodule cells (Gavrin et al., 2015). Another example is nitrate transporter, LjNPF8.6 (Phvul.006G065900), which controls the N-fixing nodule activity (Valkov et al., 2017). The circRNA (Chr06:17692671| 17693224) of the *Phvul.006G065900* gene is upregulated in the nitrogen-fixing nodule of common bean, indicating its possible role in regulation of nitrogen-fixing efficiency. Besides, GO and KEGG analysis of the circRNAs’ parental genes also showed that they may be involved in different processes or pathways during symbiosis. Our circRNA analysis suggested that circRNAs expressed in each stage may have different functions in the legume–rhizobium symbiosis.

A more complex circRNA–miRNA–mRNA network occurred in nitrogen-fixing nodules than young nodules. In both animals and plants, circRNA can bind to miRNA as competitive endogenous RNA, thereby reducing the ability of the miRNA to target mRNA and playing a key role in miRNA function and transcriptional regulation (Memczak et al., 2013; Pan et al., 2018; Wang et al., 2019). In the current study, network analysis indicated that circRNAs may play regulatory roles during nodule formation and nitrogen fixation. CircRNAs interact with miRNAs whose target genes are possibly involved in transmembrane transport, which may improve transport of various mineral nutrients during the nodule formation (Bapaume and Reinhardt, 2012). The CircRNA–miRNA–mRNA network is possibly involved in nitrogen metabolism, energy supply, and carbon–nitrogen balance, which is also essential for symbiosis during the nitrogen-fixation stage (Roy et al., 2020). In addition, the remaining 7570 circRNAs (85.6% of the total 8,842 circRNAs) without miRNA binding sites suggested that most circRNAs play other roles besides serving as miRNA sponges during nodulation and nitrogen fixation, which is also seen in animals (Guo et al., 2014).

In this study, we explore the genomic characteristics, expression profiling, regulation, and potential functions of circRNAs during the early inoculation, nodule organogenesis, and nitrogen fixation in common bean compared to the corresponding uninoculated roots. First, we identified the characteristics including genomic type, number, chromosome distribution, length distribution, and GC content of circRNAs in both developing roots and nodules. Although there were some common features in both roots and nodules, nodules produced more circRNAs than roots. Secondly, GO/KEGG analysis and other functional annotation indicated that they may play different roles in nodulation and nitrogen fixation, mainly related to oxidation–reduction, signaling, and metabolism, respectively. Thirdly, combined with our mRNA-seq for each sample, our correlation analysis revealed that most circRNAs were regulated independently of their parental genes, while the remaining minor circRNAs showed a significant positive correlation between the expression of circRNAs and their parental genes. Despite the potential role of miRNA sponges, most circRNAs did not function as miRNA sponges for post-transcriptional regulation during nodule development of common bean in our study. Our study reveals that the circRNAs may function as new regulators in legume–rhizobium symbiosis.

## Data Availability Statement

The datasets presented in this study can be found in online repositories. The names of the repository/repositories and accession number(s) can be found below: https://www.ncbi.nlm.nih.gov/, PRJNA633336.

## Author Contributions

ZW, RQ, and HoL designed and conceived the experiment. ZW, EQ, and SL analyzed the RNA-Seq data. WH and HuL performed the experiment. ZW and WH wrote the manuscript. ZW, HoL, and AG revised the manuscript. All authors contributed to the article and approved the submitted version.

## Conflict of Interest

The authors declare that the research was conducted in the absence of any commercial or financial relationships that could be construed as a potential conflict of interest.

## Supplementary Material

Supplementary Figure 1 Sampling diagram during root nodule development in common bean **(A)**, and phenotypes of root hairs inoculated with and without rhizobia at 1 day after inoculation (DAI) **(B)**. 1 DAI, 10 DAI, and 21 DAI represent three stages: early inoculation, nodule organogenesis, and nitrogen fixation. “I” and “C” represent the samples inoculated with and without rhizobia, respectively.

Supplementary Figure 2 Characteristics of circRNAs in root nodules of common bean. **(A)** Principal component analysis of 18 samples with circRNA-seq. **(B)** GC content distribution analysis of circRNAs. **(C)** Chromosome distribution analysis of circRNAs.

Supplementary Figure 3 Differential expression analysis of circRNAs during nodule development. **(A)** Venn diagram of differentially expressed circRNAs during nodule organogenesis. **(B)** Clustering heat map of differentially expressed circRNAs during nodule organogenesis. **(C)** Venn diagram of differentially expressed circRNAs during nitrogen fixation. **(D)** Clustering heat map of differentially expressed circRNAs during nitrogen fixation.

Supplementary Figure 4 KEGG pathway analysis of upregulated DEcircRNAs during nodule development. **(A)** KEGG pathway analysis of 32 upregulated DEcircRNAs during nodule organogenesis. **(B)** KEGG pathway analysis of 143 upregulated DEcircRNAs during nitrogen fixation.

Supplementary Figure 5 Expression profiles of circRNAs and mRNA during nodule development. **(A)** Clustering heat map of differentially expressed circRNAs during root nodule development. **(B)** Clustering heat map of differentially expressed mRNAs during root nodule development.

Supplementary Table 1 Statistics of reads from 18 circRNA-seq samples.

Supplementary Table 2 Identified 8,842 circRNAs with high confidence in six groups and their corresponding parental genes. “0” and “1” represent the absence or presence in each condition, respectively.

Supplementary Table 3 18 divergent primers and 18 convergent primers for validation for circRNA genuineness.

Supplementary Table 4 16 divergent qRT-PCR primers for quantification of circRNA expression.

Supplementary Table 5 The targeted binding relationships between 1,272 circRNAs and 229 miRNAs.

Supplementary Table 6 The inferred circRNA-miRNA-mRNA regulatory relationships during nodule organogenesis.

Supplementary Table 7 GO terms of miRNA target genes during nodule organogenesis.

Supplementary Table 8 KEGG pathways of miRNA target genes during nodule organogenesis.

Supplementary Table 9 The inferred circRNA-miRNA-mRNA regulatory relationships during nitrogen fixation.

Supplementary Table 10 GO terms of miRNA target genes during nitrogen fixation.

Supplementary Table 11 KEGG pathways of miRNA target genes during nitrogen fixation.
