# Supplementary figures and images for: Comprehensive Identification and Expression Profiling of Circular RNAs During Nodule Development in Phaseolus vulgaris
Source: Front Plant Sci. 2020 Oct 28;11:587185. doi: 10.3389/fpls.2020.587185 (PMC7655914; doi:10.3389/fpls.2020.587185)

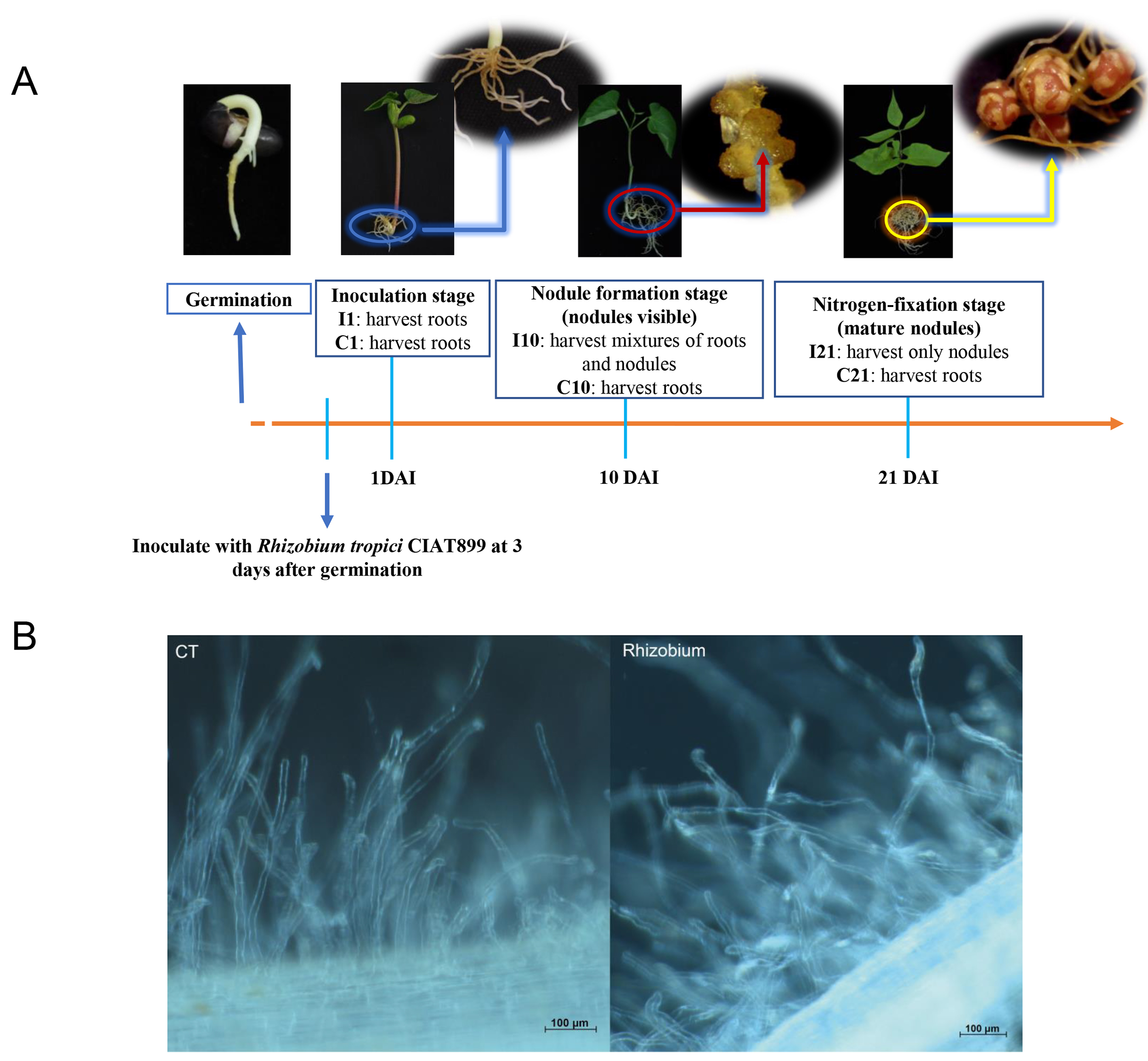

Supplement: Supplementary Figure 1 — Sampling diagram during root nodule development in common bean (A), and phenotypes of root hairs inoculated with and without rhizobia at 1 day after inoculation (DAI) (B). 1 DAI, 10 DAI, and 21 DAI represent three stages: early inoculation, nodule organogenesis, and nitrogen fixation. “I” and “C” represent the samples inoculated with and without rhizobia, respectively. [file Image_1.JPEG]

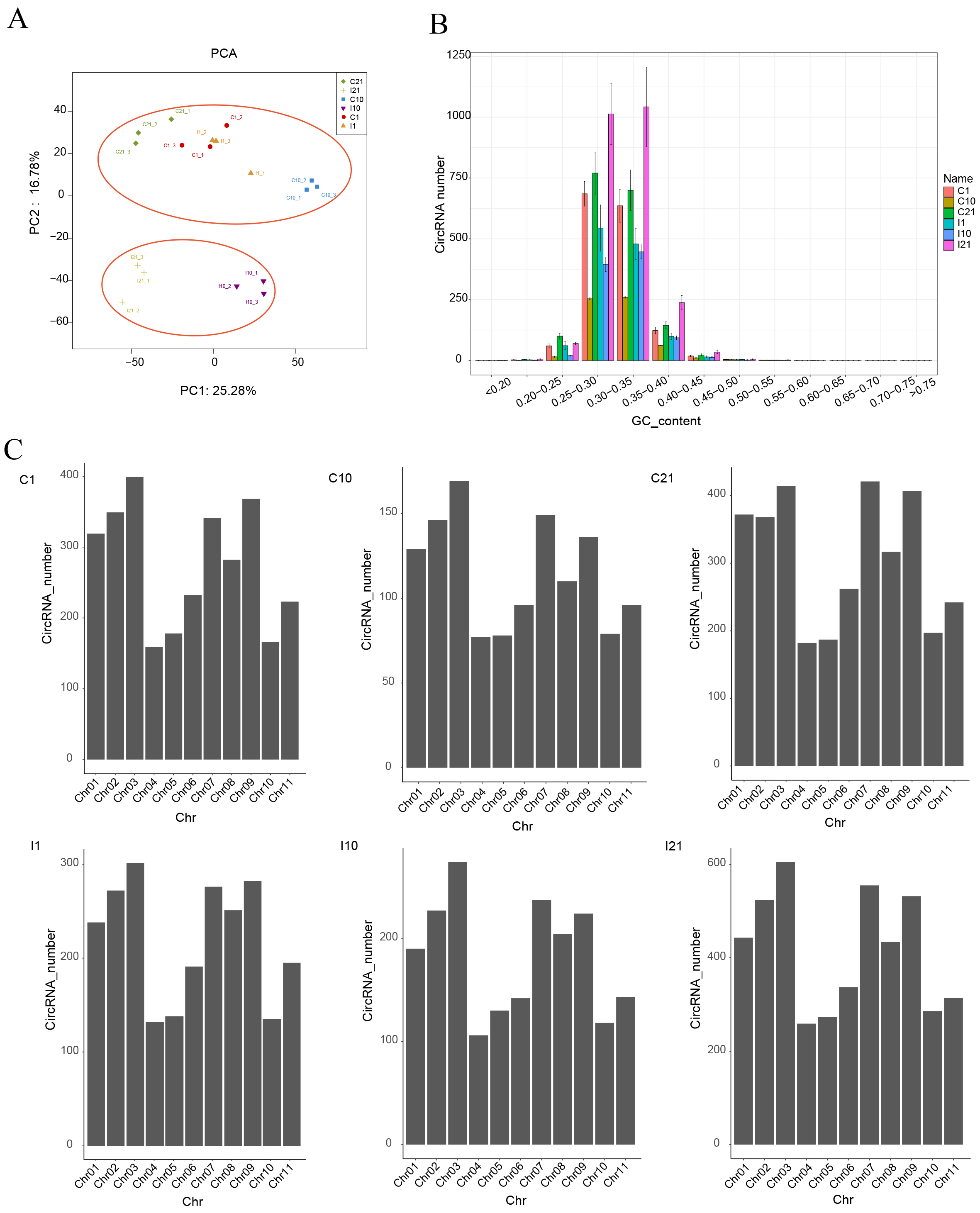

Supplement: Supplementary Figure 2 — Characteristics of circRNAs in root nodules of common bean. (A) Principal component analysis of 18 samples with circRNA-seq. (B) GC content distribution analysis of circRNAs. (C) Chromosome distribution analysis of circRNAs. [file Image_2.JPEG]

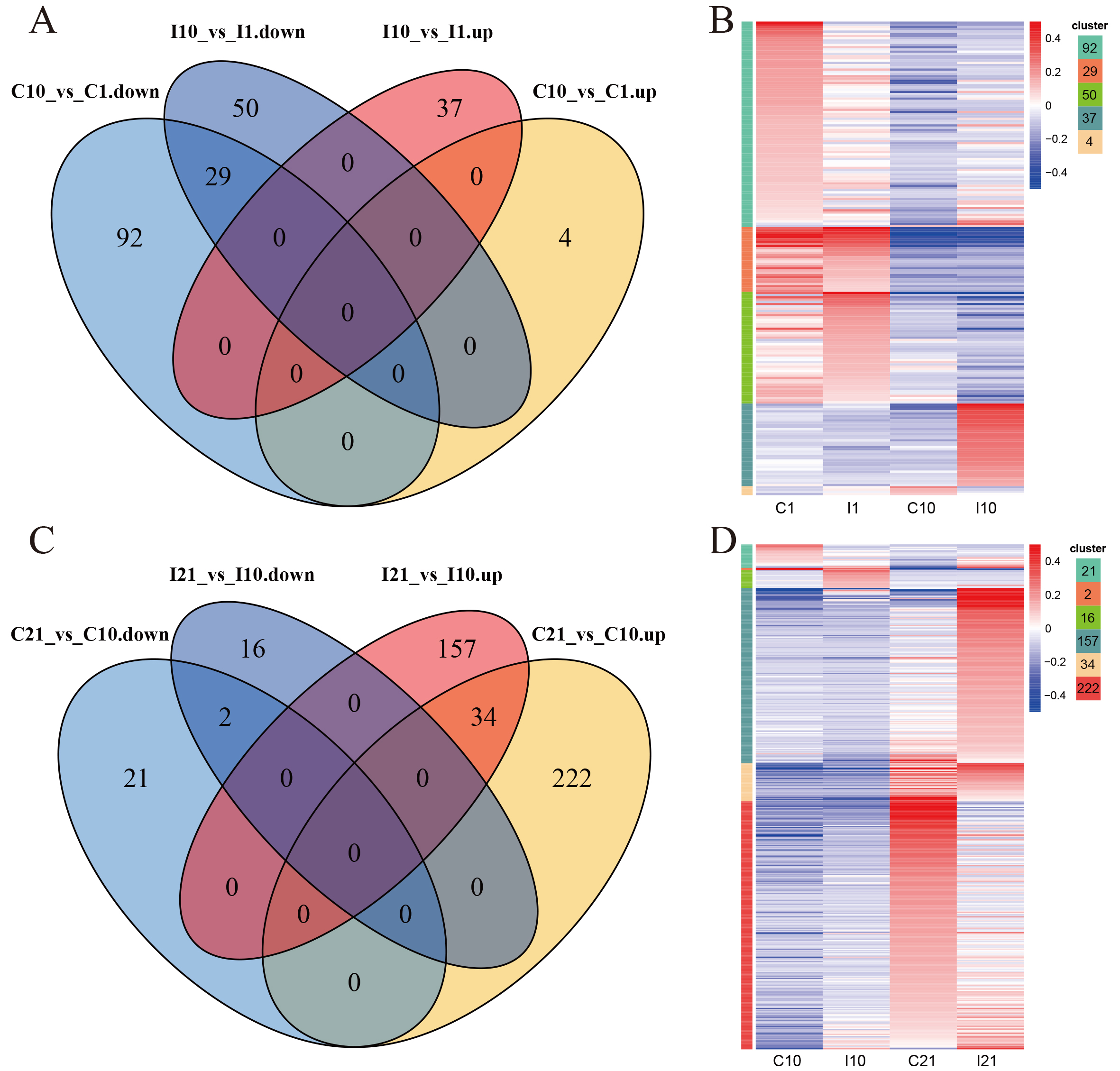

Supplement: Supplementary Figure 3 — Differential expression analysis of circRNAs during nodule development. (A) Venn diagram of differentially expressed circRNAs during nodule organogenesis. (B) Clustering heat map of differentially expressed circRNAs during nodule organogenesis. (C) Venn diagram of differentially expressed circRNAs during nitrogen fixation. (D) Clustering heat map of differentially expressed circRNAs during nitrogen fixation. [file Image_3.JPEG]

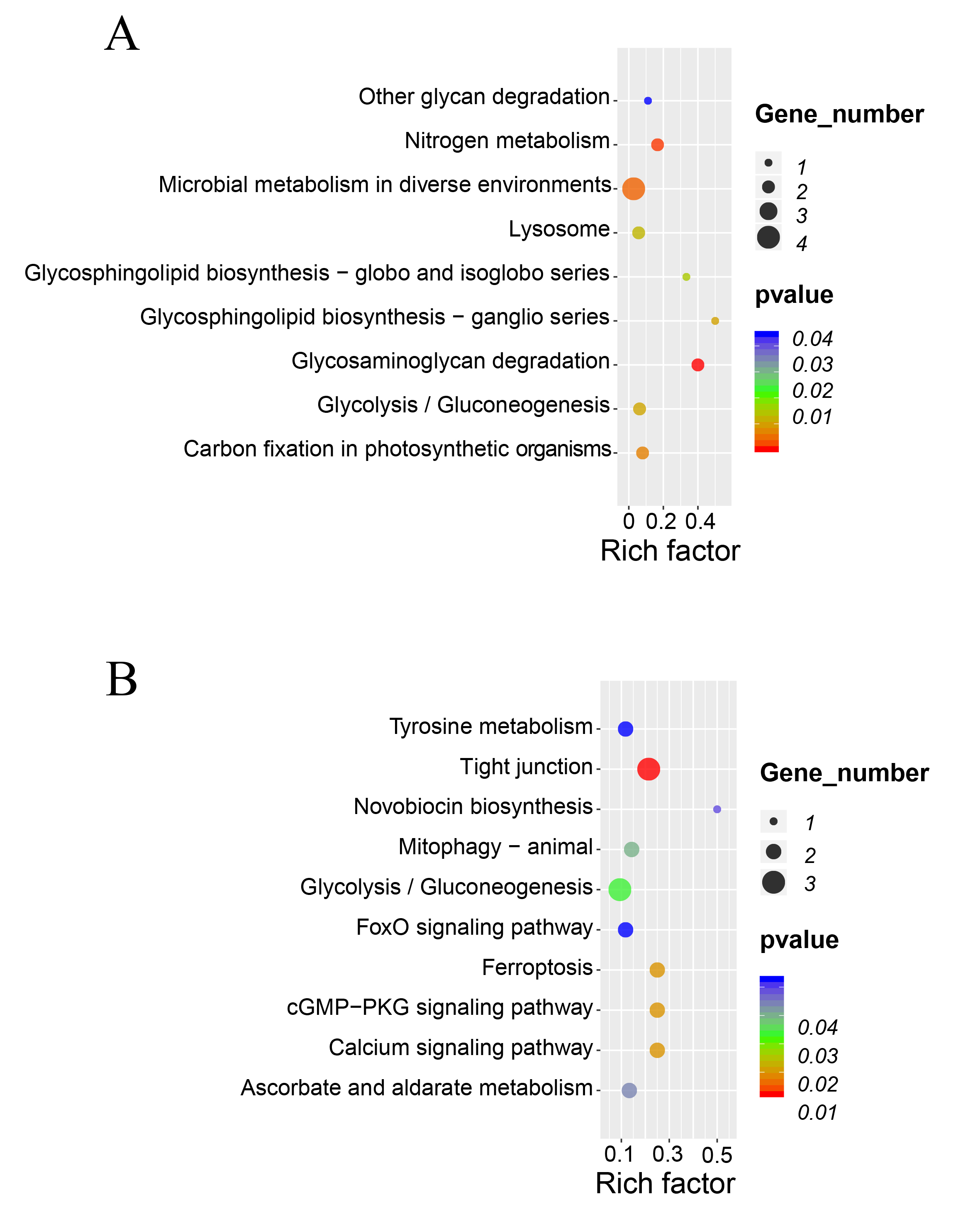

Supplement: Supplementary Figure 4 — KEGG pathway analysis of upregulated DEcircRNAs during nodule development. (A) KEGG pathway analysis of 32 upregulated DEcircRNAs during nodule organogenesis. (B) KEGG pathway analysis of 143 upregulated DEcircRNAs during nitrogen fixation. [file Image_4.JPEG]

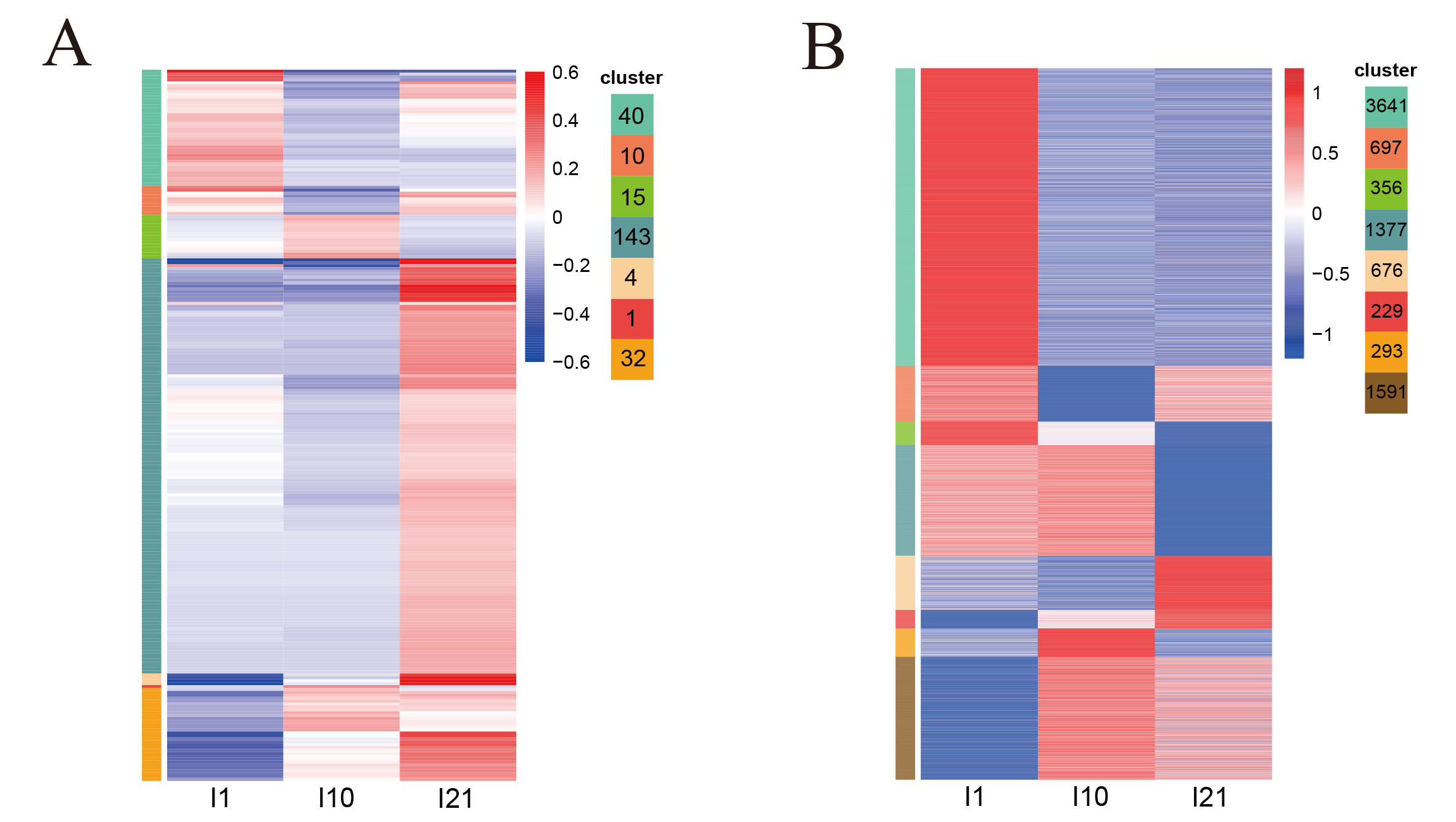

Supplement: Supplementary Figure 5 — Expression profiles of circRNAs and mRNA during nodule development. (A) Clustering heat map of differentially expressed circRNAs during root nodule development. (B) Clustering heat map of differentially expressed mRNAs during root nodule development. [file Image_5.JPEG]
